# Cognitive demands during quiet standing elicit truncal tremor in two frequency bands: differential relations to tissue integrity of corticospinal tracts and cortical targets

**DOI:** 10.3389/fnhum.2015.00175

**Published:** 2015-04-07

**Authors:** Edith V. Sullivan, Natalie M. Zahr, Torsten Rohlfing, Adolf Pfefferbaum

**Affiliations:** ^1^Alcohol Translational Neuroscience Program, Department of Psychiatry and Behavioral Sciences, Stanford University School of MedicineStanford, CA, USA; ^2^Neuroscience Program, SRI InternationalMenlo Park, CA, USA

**Keywords:** diffusion tensor imaging, cerebellum, motor cortex, tremor, quiet standing, cognitive demands, corticospinal tract

## Abstract

The ability to stand quietly is disturbed by degradation of cerebellar systems. Given the complexity of sensorimotor integration invoked to maintain upright posture, the integrity of supratentorial brain structures may also contribute to quiet standing and consequently be vulnerable to interference from cognitive challenges. As cerebellar system disruption is a common concomitant of alcoholism, we examined 46 alcoholics and 43 controls with a force platform to derive physiological indices of quiet standing during cognitive (solving simple, mental arithmetic problems) and visual (eyes closed) challenges. Also tested were relations between tremor velocity and regional gray matter and white matter tissue quality measured with the diffusion tensor imaging (DTI) metric of mean diffusivity (MD), indexing disorganized microstructure. Spectral analysis of sway revealed greater tremor in alcoholic men than alcoholic women or controls. Cognitive dual-tasking elicited excessive tremor in two frequency bands, each related to DTI signs of degradation in separate brain systems: tremor velocity at a low frequency (2–5 Hz/0–2 Hz) correlated with higher MD in the cerebellar hemispheres and superior cingulate bundles, whereas tremor velocity at a higher frequency (5–7 Hz) correlated with higher MD in the motor cortex and internal capsule. These brain sites may represent “tremorgenic networks” that, when disturbed by disease and exacerbated by cognitive dual-tasking, contribute to postural instability, putting affected individuals at heightened risk for falling.

## Introduction

Tremor in alcohol-dependent individuals observed clinically is typically the result of acute withdrawal from alcohol and most obviously exhibited as “the shakes” (e.g., Neiman et al., 1990). Beyond delirium tremens, tremor can persist with extended sobriety (Koller et al., 1985). Postural tremor elicited during quiet standing, initially described by Holmes (1917), has been attributed to damage of the cerebellum and its efferent circuitry and was described physiologically by involuntary movement in the 3–10 Hz frequency band in case studies of patients with known or presumed cerebellar lesions (e.g., Brown et al., 1997). Among such patients are chronic alcoholics, who as a group, can exhibit cellular damage of cerebellar vermis postmortem (e.g., Victor et al., 1989; Pentney, 1993; Baker et al., 1999; Harper et al., 2003) and vermian and cerebellar hemisphere shrinkage *in vivo* (e.g., Sullivan et al., 2000, 2006; Makris et al., 2008; Le Berre et al., 2014).

Consistent with those case studies, truncal tremor during quiet standing, measured by analyzing the temporal frequency of sway paths derived from a force platform, was detected in the 2–5 Hz frequency band and the 5–7 Hz band in alcoholic men (Sullivan et al., 2006) and women (Sullivan et al., 2010) tested well beyond acute withdrawal. Both sway and tremor could be quelled by introducing sensory and motor-stabilizing factors of vision, touch, and broad-based stance, even though the magnitude of sway and 5–7 Hz tremor were related to local CNS integrity, i.e., volume shrinkage of the anterior vermis (Sullivan et al., 2006, 2010). Although some factors can quell sway and tremor, it remains untested whether engaging in a cognitively challenging task known to impair stability (Pellecchia, 2003) can promote instability or tremor during quiet standing in recovering alcoholics and whether the extent of such disruption would be associated with tissue integrity of cerebellar structures or cortical regions.

Maintenance of upright posture requires complex sensorimotor integration (Diener and Dichgans, 1992; Baloh et al., 1998). Thus, the ability to stand quietly can potentially be impaired by engagement in competing cognitive tasks that share attentional resources with motor stability systems (Shumway-Cook et al., 1997; Pellecchia and Turvey, 2001). Specifically, engagement in difficult compared with simple working memory tasks resulted in greater difficulty in controlling postural sway (Pellecchia, 2003) and, in certain situations, with increasing rigidity while attempting to reduce sway (Dault et al., 2003). That cognitive tasks can interact with postural stability suggests that neural sources of these actions and interactions involve both cerebellar and supratentorial brain motor and cognitive systems, especially ones that share connections. This possibility was borne out in a recent experiment using functional near infrared spectroscopy (fNIRS) acquired while participants were standing still or walking and while engaged in a simple cognitive task (counting forward) or a complex one (serially subtracting 7 s) (Mirelman et al., 2014). Frontal cortical brain activation showed a graded effect, being greatest under the most challenging condition of walking while doing serial 7 s, less so when standing while doing serial 7 s, and negligible when standing or walking while counting forward. Thus, articulation alone was inadequate to invoke frontal activation when engaged in motor-cognitive dual tasking.

Relations between abnormally hyperintense magnetic resonance imaging (MRI) signal of nonselective supratentorial white matter and falling in the elderly are well established (for review Zheng et al., 2011). Evidence for the role of extensive circuitry in tremor production derives from observations of relief of essential tremor by application of repetitive transmagnetic stimulation over the motor cortex (Rogasch and Todd, 2013). Particularly convincing are results from a study combining deep brain stimulation (DBS) of the ventral thalamus for relief of intractable tremor and fiber tracking of diffusion tensor imaging (DTI) data. That study used the locus inducing successful relief as the seed point for fiber tracking of DTI data acquired in the patients. In addition to confirming the relevance of connectivity between the ventral thalamus and primary motor cortex to successful DBS outcome, structural connectivity sites included precentral gyrus, supplementary motor area, frontal gyri, anterior cingulum, and the cerebellar hemispheres at the level of the superior cerebellar peduncles (Klein et al., 2012).

Here, we sought to examine relations among quantitative assessment of quiet standing on a force platform in alcoholic men and women with extended sobriety compared with age- and sex-matched controls. Path length and frequency data were acquired while participants engaged in cognitive challenges of increasing difficulty related to mental arithmetic and visual input and were evaluated for their relation with measures of brain white matter fiber tract and gray matter tissue integrity using mean diffusivity (MD) measures from DTI potentially contributing to postural stability. The white matter systems examined were the internal capsule, pontocerebellar tract, genu and splenium of the corpus callosum, three segments of the cingulate bundle; the gray matter nodes examined were the precentral, superior, middle, and inferior frontal cortical gray matter, and thalamus, Crus I and II, and anterior, posterior, and inferior sectors of the vermis. MD was chosen over other DTI metrics, including anisotropy, because of its sensitivity to detection of alcoholism-related brain deficits and demonstration of brain-behavior relations (Pfefferbaum et al., 2006, 2009) and because MD is more readily interpretable than anisotropy as an index of gray matter tissue integrity.

## Materials and methods

### Participants

The groups comprised 46 alcoholics and 43 controls age-range matched to the alcoholics. All participants gave written informed consent for participation in this study, which was approved by the Internal Review Boards of Stanford University School of Medicine and SRI International; these boards assured that our study was conducted in accordance with the Declaration of Helsinki. Although the alcoholics had fewer years of education and were of lower socioeconomic status (Hollingshead, 1975) than the controls, the groups did not differ substantially in intelligence estimated with the National Adult Reading Test (NART) (Nelson, 1982), Dementia Rating Scale (Mattis, 2004), handedness (Crovitz and Zener, 1962), or body mass index (Table 1). The alcoholic men and women had a higher incidence of family history of alcoholism than control men and women. Although the alcoholic men had greater lifetime consumption of alcohol than alcoholic women, neither age of alcoholism onset nor median number of days since their last drink differed significantly between the sexes. Three alcoholic men and one alcoholic woman reported drinking within a week of testing; none demonstrated withdrawal symptoms.

**Table 1:** **Group demographics: mean (±SD)**.

	**Men**		**Women**		**ANOVA or χ^2^*p*-value**
	**Control (CM)**	**Alcoholic (AM)**	**Control (CF)**	**Alcoholic (AF)**	**Scheffe paired tests**
N with balance data	23	32	20	14	
N with balance+DTI data	18	30	20	12	
Age (years)	43.2 (11.0)	47.6 (10.5)	40.9 (10.8)	49.0 (10.9)	0.079 n.s. by Scheffe
Education (years)	15.2 (1.8)	13.7 (2.5)	15.8 (2.2)	13.4 (1.7)	0.001 CM = CF > AM = AF
NART IQ	111.9 (7.6)	106.5 (9.7)	112.7 (9.0)	107.3 (9.6)	0.047 n.s. by Scheffe
Dementia Rating Scale^†^	140.2 (2.8)	138.2 (4.3)	140.1 (2.2)	138.3 (3.6)	0.078 n.s. by Scheffe
Socioeconomic status^††^	30.9 (11.6)	36.7 (12.7)	28.4 (11.7)	39.6 (11.0)	0.002 CF > AM
Handedness^†††^	25.9 (11.7)	25.7 (14.2)	22.9 (9.2)	22.0 (6.8)	0.663 n.s. by Scheffe
Family history of alcoholism negative/positive	16/5	17/3	11/21	5/9	0.0003 CM = CF ≠ AM=AF
Body mass index	26.4 (4.0)	27.1 (4.2)	25.1 (5.7)	25.5 (5.2)	0.448 n.s. by Scheffe
Lifetime alcohol consumption (kg)	29.5 (62.5)	1309.7 (937.2)	11.3 (12.0)	831.5 (743.8)	0.0001 CM = CF < AM = AF
Alcoholism onset age (years);	—	21.7 (7.4);	—	25.3 (8.8)	0.159 n.s. by Scheffe
Median days since last drink (range);	—	136.3 (2–2296);	—	162.0 (3–2553)	Mann-Whitney *U*-test 0.3765

† *DRS range = 127–144; dementia cut-off < 124*.

†† *Lower scores signify higher status*.

††† *Right handedness = 14–32; left handedness = 50–70*.

### Quiet standing quantified with force platform analysis

#### Test conditions

Subjects wore standard rubber-soled socks and stood still on a force plate with feet together with arms relaxed at their sides. Each of three conditions, comprising one easy and one hard arithmetic condition and a no-task control, was performed with eyes open and with eyes closed, producing six test conditions. A camera monitored compliance with eyes-closed instruction. In the two arithmetic conditions, subjects listened to sets of 9, pre-recorded digit strings, broadcast through speakers mounted on each side of the force platform. Number strings were spoken in 4.5 s epochs, followed by 0.5 s intervals for responding (50 s = 2 s before a trial+45 s of math+3 s intertrial interval). In the two easy conditions, subjects heard three digits and two operands followed by a correct (66%) or incorrect (33%) answer (e.g., 2 + 5−3 = 4; 3−1 + 8 = 11) to which they said “yes” if correct or “no” if incorrect. In the two hard conditions, subjects additionally responded “odd” or “even” to classify the given answer. Before testing, subjects completed practice trials of the arithmetic problems while seated.

#### Sway path analysis

Balance was assessed with a microcomputer-controlled force plate (model 9284; Kistler, Amherst, NY) with multiple transducers and analog-digital converters. Data were sampled at 1000 Hz; raw data were 45 s continuous trials of center-of-pressure displacements (x-y pairs). Data were subjected to a 10 Hz lowpass filter (99 terms, −50 db Gibbs); sway path length (P) was expressed as the line integral (cm):

$$\text{P}=\sum_{i\;=\;1}^{N-1}\sqrt{{\left(x_{i\;+\;1}-x_{i}\right)}^{2}\;+\;{\left(y_{i\;+\;1}-y_{i}\right)}^{2}}.$$

#### Tremor analysis

Frequency analyses used fast Fourier transform on the anterior-posterior and lateral-medial sway path velocity (2-point differential of the filtered sway path). To characterize the frequency (Hz) of maximal sway velocity, data were divided into three frequency components: 0–2, 2–5, and 5–7 Hz. Following the method of Baloh et al. (1998) to characterize the frequency (Hz) of maximal sway velocity selective to patients with cerebellar damage, we derived a frequency quotient, which was the power of the spectral frequencies between 2 and 5 Hz divided by those between 0 and <2 Hz, with the expectation that the alcoholics would have a higher ratio than control subjects. Further, only velocity was analyzed because prior work had shown that frequency analysis of center-of-pressure sway-path velocity (distance/time) to be more sensitive than amplitude (distance) (Baloh et al., 1998).

### Sensory testing

Most participants underwent sensory testing of the lower extremities to assess the contribution of cutaneous and other peripheral sensory impairment on metrics of quiet standing. Deep Tendon Reflex, Great Toe Vibration, and 2-point Discrimination are standard neurological tests of the lower limbs (Bigley, 1990).

#### Subjective assessment of peripheral neuropathy

Subjects were asked to rate on a 0–10 point scale discomfort (pain, aching, burning; pins and needles; numbness) in feet or legs that might by symptomatic of peripheral neuropathy.

#### Deep Tendon Reflex

The subject sat on an examination table. The examiner used one hand to press upward on ball of foot (dominant side first) dorsiflexing the subject's ankle to 90° and then striking the Achilles tendon with a reflex hammer. Reflexes were felt by examiner's hand as plantar flexions. Deep Tendon Reflex can discern *hyporeflexia*, an absent or diminished response to tapping, typically indicating disease involving one or more of the components of the reflex arc or *hyperreflexia*, hyperactive or repeating (clonic) reflexes, indicating an interruption of corticospinal and other descending pathways that influence the reflex arc.

#### Perception of great toe vibration

The subject lay supine on the examination table. The examiner then struck a tuning fork and placed it on distal interphalangeal joint of the great toe on the dominant side and asked when the vibration stopped. The time from touch to response was timed with a stopwatch. The procedure was repeated on the other great toe. The timed vibratory test is the most sensitive noninvasive method of detecting mild to moderate impairments in vibratory sensation and used to detect sensory neuropathy (Cherry et al., 2005).

#### 2-point discrimination

Using a 3-point aesthesiometer, the examiner touched the sole of one foot with 1 or 2 points, to which the subject (with eyes closed) responded “one” or “two.” Testing avoided calloused skin and proceeded with descending limits (starting at 50 mm distance between points); the threshold was the shortest distance on which fewer than 3 errors were made (Corkin et al., 1970). The procedure was repeated with the other foot.

### MRI and DTI protocol and analysis

Neuroimaging and balance data of the alcoholics (30 men and 12 women) and most controls (18 men and 20 women) were collected either on the same day or within 1 week of each other. Exceptions were three control men with multiple MRIs, for whom we chose the MRI data set closest to the balance date (1–2.5 years difference).

#### DTI and MRI acquisition

Imaging data were acquired on a General Electric 3T clinical human MR system with an 8-channel head coil after higher-order (nonlinear) shimming (Kim et al., 2002). DTI and fast spin-echo (FSE) structural data were collected with the same slice locations: DTI (2D echo-planar, axial plane, *TR* = 7300 ms, *TE* = 86.6 ms, thickness = 2.5 mm, skip = 0 mm, locations = 62, *b* = 0 (5 NEX) + 15 noncollinear diffusion directions *b* = 860 s/mm^2^ (2 NEX)+15 opposite-polarity noncollinear diffusion directions *b* = 860 s/mm^2^ (2 NEX), FOV = 240 mm, x-dim = 96, y-dim = 96, reconstructed to 128 × 128, 4030 total images); FSE (2D axial, *TR* = 7850 ms, *TE* = 17/102 ms, thickness = 2.5 mm, skip = 0 mm, locations = 62). An aligned T1-weighted SPGR (3D axial IR-prep, *TR* = 6.5 ms, *TE* = 1.6 ms, thick = 1.25 mm, skip = 0 mm, locations = 124) was collected, such that two 1.25 mm SPGR slices subtended each 2.5 mm-thick FSE/DTI slice. A field-map was generated from a gradient recalled echo sequence pair (*TR* = 460 ms, *TE* = 3/5 ms, thickness = 2.5 mm, skip = 0 mm, locations = 62).

#### DTI analysis

DTI quantification was preceded by eddy-current correction on a slice-by-slice basis using within-slice registration. B_0_-field inhomogeneity-induced geometric distortion in the eddy current-corrected images was corrected with PRELUDE [Phase Region Expanding Labeller for Unwrapping Discrete Estimates (Jenkinson, 2003)] and FUGUE [FMRIB's Utility for Geometrically Unwarping EPIs (Jenkinson and Smith, 2001)].

#### Fiber bundle identification and tracking

Fiber tracking was performed with software by Gerig et al. (2005) based on the “target” and “source” method of Mori and Van Zijl (2002). In short, streamlines originate from each pixel in a “source” region and follow the directions of the principal eigenvectors of the diffusion tensors until either the curvature exceeds a maximum angle or the local fractional anisotropy (FA) falls below a threshold. Tracking parameters included white matter extraction threshold (minimum FA) of 0.17, fiber tracking threshold of 0.125, and maximum voxel-to-voxel coherence minimum transition smoothness threshold of 0.80 (~37° maximum deviation between voxels), with essentially no limit on the number of fibers. Only streamlines that also traverse at least one pixel of a “target” region are kept; all other streamlines are discarded. Targets and sources were identified on the FA image of the SRI24 atlas (Rohlfing et al., 2010) (http://nitrc.org/projects/sri24) and mapped to the corresponding locations on the native-space DTI images for each subject using coordinate transformations computed by nonrigid image registration (Rohlfing and Maurer, 2003) (http://nitrc.org/projects/cmtk/). The output of the fiber tracking for each subject and each source-target pair was a 3D geometric model of the fiber paths comprising a table of all point locations along each fiber with local DTI metrics; mean diffusivity (MD) for each fiber bundle was the unit of analysis.

#### Gray matter and tissue mean diffusivity (MD) analysis

The T1-weighted SPGR images were registered nonrigidly to the T1 image of the SRI24 atlas, and tissue probability maps transferred from the atlas to each SPGR image. Using these probabilities to both initialize and guide tissue classification, the SPGR images were then segmented into CSF, gray matter, white matter, or tissue (gray+white matter) using FSL's FAST tool (Zhang et al., 2001). Furthermore, by transferring label maps from the SRI24 atlas to each SPGR image, the latter was parcellated into cortical and subcortical regions of interest. Selected regions were also mapped, again via a nonrigid registration to the corresponding locations on the native-space MD images providing an index of tissue quality, with higher gray matter MD indicative of greater presence of interstitial fluid.

### Statistical analysis

Group and condition effects for the primary balance measures of sway path length and tremor frequency (2–5/0–2 Hz and 5–7 Hz) were tested with repeated-measures analysis of variance (ANOVA) for 4 groups (control and alcoholic men and women), two vision conditions (eyes open, eyes closed), two sway or tremor directions (anterior-posterior, lateral-medial), and three task conditions (no, easy, hard), with Geiser-Greenhouse (GG) correction where appropriate. Bivariate and multiple regression analyses examined relations between and among variables and follow up with calculations of confidence intervals based on bootstrapping computations based on 2000 permutations. To reduce the number of comparisons, correlations were based on anterior/posterior (AP)—medial/lateral (ML) difference measures (i.e., AP–ML) of sway path length and tremor velocity.

## Results

### Measures of quiet standing

#### Sway path length

**Figure 1:**
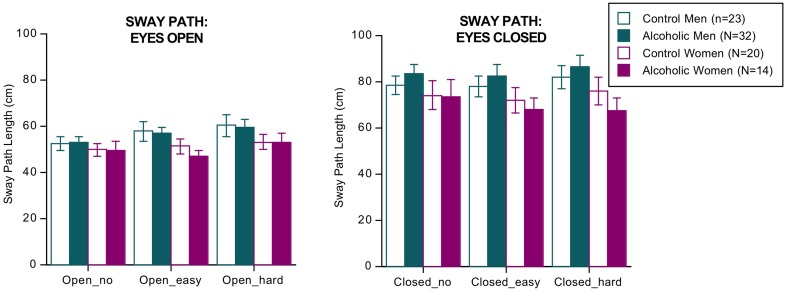
**Bar graphs = mean ± SE of the sway path length in each group**. None of the group differences was significant.

ANOVA (4 groups × 2 eyes open/closed conditions × 3 dual tasks conditions of no, easy, and hard arithmetic × 2 sway directions [AP, ML]) identified three significant effects and two interactions but no significant group differences or interactions involving group (Figure 1). The significant effects indicated that, regardless of group, sway paths were longer under the following conditions: with eyes closed than open [*F*_(1, 85)_ = 268.144, *p* = 0.0001 GG], with hard relative to easy arithmetic dual task [*F*_(2, 170)_ = 5.039, *p* = 0.0121 GG], and in the AP relative to the ML direction [*F*_(1, 85)_ = 4.026, *p* = 0.0480 GG]. The significant interactions involved vision × task [*F*_(2, 170)_ = 4.037, *p* = 0.0212 GG] and vision x task x direction [*F*_(2, 170)_ = 9.239, *p* = 0.0002 GG].

The groups did not differ in number of errors committed on the arithmetic task, and in no condition did sway path length (based on the AP–ML difference) correlate with number of arithmetic errors by the alcoholic men nor with body mass index. The results did not change when recalculated without the four alcoholics who had drunk within a week of testing.

#### Tremor

**Figure 2:**
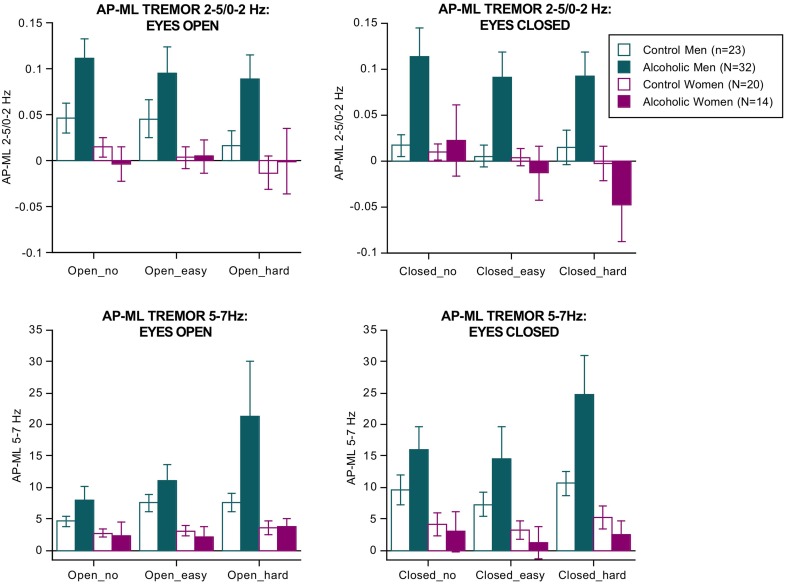
**Bar graphs = mean ± SE of the 2–5/0–2 Hz (top) and 5–7 Hz frequency bands expressed as the difference between the anterior-posterior tremor velocity minus the medial-lateral tremor velocity in each group**. Tremor was greatest in the alcoholic men; 5–7 Hz tremor velocity showed a step-wise increase with increasing difficulty of the dual task.

Separate ANOVAs were conducted for the two tremor bands (Figure 2). For 2–5/0–2 Hz, the effects of group [*F*_(3, 85)_ = 3.188, *p* = 0.0278], vision [*F*_(1, 85)_ = 15.198, *p* = 0.0002], direction [*F*_(1, 85)_ = 18.826, *p* = 0.0001], group × direction interaction (3, 85) = 6.359, *p* = 0.0006 GG), and dual task × direction interaction [*F*_(2, 170)_ = 5.800, *p* = 0.0038 GG] were significant.

For the 5–7 Hz band, the effects of group [*F*_(3, 85)_ = 3.472, *p* = 0.0196], vision [*F*_(1, 85)_ = 63.288, *p* = 0.0001], and dual task [*F*_(2, 170)_ = 12.890, *p* = 0.0001 GG] were significant. Also significant were the group × direction [*F*_(3,85)_ = 5.142, *p* = 0.0026 GG] and the group × direction-dual task [*F*_(6, 170)_ = 2.895, *p* = 0193 GG] interactions. These effects indicate that the alcoholic men exhibited by far the greatest tremor in both frequency bands of any group, especially in the AP direction, and showed a modest increase in tremor with increasing task difficulty regardless of visual condition (Figure 2). These findings endured when the 3 alcoholic men who had drunk alcohol within a week of testing were excluded from analysis.

In the alcoholic men, greater AP-ML tremor velocity occurred with fewer arithmetic errors and was significant for the 2–5/0–2 Hz ratio with eyes open (easy Rho= −0.43, *p* = 0.0245; hard Rho = −0.421, *p* = 0.0285) and for the 5–7 Hz band with eyes closed (easy Rho = −0.47, *p* = 0.0143; hard Rho = −0.385, *p* = 0.0455). Path length (AP–ML, eyes open, hard arithmetic) correlated with tremor velocity only in the 5–7 Hz band (Rho = 0.43, *p* = 0.0215). In no condition did tremor velocity in either frequency band correlate with body mass index or length of sobriety.

### Sensory testing

Subjective lower limb discomfort was rated none to mild: rating = 1 by one alcoholic man and 3 by one alcoholic woman. The only objective measures on which the groups differed were vibration perception of the left great toe (Kruskal-Wallis *H* = 10.16, *p* = 0.0173) and two-point discrimination (Kruskal-Wallis *H* = 16.30, left foot *p* = 0.001, *H* = 12.34 right foot *p* = 0.0063; 1 subject's values >11 SD and were Windsorized to the next highest value; thus left foot value moved from 45 to 25, and right foot value moved from 40 to 24). For both objective tests, only the alcoholic men had abnormally high scores. Correlations conducted between the mean of the left+right foot 2-point discrimination scores and the balance measures identified one modest correlation, which was with tremor velocity in the 2–5/0–2 Hz frequency band in the eyes-open condition (*r* = 0.40, *p* = 0.0415; Rho = 0.41, *p* = 0.0402).

### Fiber tracking and gray matter MD correlates of sway path length and tremor

White matter fiber bundle correlates of balance measures evaluated were the internal capsule, pontocerebellar tract, genu and splenium of the corpus callosum, and three segments of the cingulate bundle. Diffusivity correlates were the precentral, superior, middle, and inferior frontal cortical gray matter and tissue (gray matter+white matter) of the thalamus, cerebellar Crus I and II, and anterior, posterior, and inferior sectors of the vermis. Although the groups did not differ in MD in these regions or fiber tracts, greater tremor correlated with higher MD in a number of regions in the alcoholic men (Table 2).

**Table 2:** **Pearson correlations between balance metric (eyes closed) and DTI diffusivity**.

**30 Alcoholic Men**		**Eyes closed/no arithmetic**			**Eyes closed/easy arithmetic**			**Eyes closed/hard arithmetic**		
		**Path length**	**2–5/0–2 Hz**	**5–7 Hz**	**Path length**	**2–5/0–2 Hz**	**5–7 Hz**	**Path length**	**2–5/0–2 Hz**	**5–7 Hz**
**FIBER BUNDLE DIFFUSIVITY**										
Internal capsule	*r*=	−0.002	0.329	0.364	0.242	0.321	0.500	0.403	0.443	**0.668**
	*p*=	0.993	0.076	0.048	0.198	0.083	0.005	0.027	0.014	0.0001^*^
Pontocerebellar tract	*r*=	−0.083	0.044	0.093	0.057	−0.175	0.239	0.199	−0.015	0.240
	*p*=	0.663	0.819	0.624	0.765	0.355	0.203	0.291	0.939	0.202
Callosal genu	*r*=	−0.126	0.177	0.208	0.099	0.213	0.311	0.194	0.215	0.445
	*p*=	0.506	0.349	0.271	0.603	0.258	0.095	0.304	0.255	0.014
Callosal splenium	*r*=	0.019	0.309	0.389	0.206	0.210	0.417	0.287	0.241	**0.547**
	*p*=	0.922	0.096	0.034	0.276	0.265	0.022	0.124	0.200	0.0017^*^
Superior cingulum	*r*=	0.026	0.419	0.327	0.145	0.369	0.385	0.341	**0.529**	**0.625**
	*p*=	0.892	0.021	0.077	0.446	0.045	0.036	0.065	**0.0027**	0.0002^*^
Posterior cingulum	*r*=	0.073	0.150	0.227	0.147	0.254	0.162	0.324	0.348	0.323
	*p*=	0.701	0.428	0.228	0.440	0.175	0.393	0.081	0.059	0.081
Inferior cingulum	*r*=	−0.112	0.101	0.219	0.013	0.327	0.051	0.133	0.232	0.234
	*p*=	0.556	0.596	0.244	0.947	0.078	0.789	0.483	0.218	0.213
**GRAY MATTER DIFFUSIVITY**										
Precentral gyrus	*r*=	0.171	0.422	0.294	0.309	0.165	0.482	0.337	0.409	**0.624**
	*p*=	0.366	0.020	0.114	0.096	0.383	0.007	0.069	0.025	0.0002^*^
Superior frontal gyrus	*r*=	0.057	0.245	0.188	0.231	0.271	0.318	0.297	0.358	**0.608**
	*p*=	0.765	0.193	0.320	0.220	0.147	0.087	0.111	0.052	**0.0004**
Middle frontal gyrus	*r*=	0.085	0.317	0.225	0.296	0.180	0.389	0.314	0.333	**0.605**
	*p*=	0.655	0.088	0.232	0.112	0.342	0.034	0.091	0.072	0.0004^*^
Inferior frontal gyrus	*r*=	0.112	0.280	0.227	0.306	0.212	0.406	0.321	0.328	**0.591**
	*p*=	0.556	0.134	0.229	0.100	0.261	0.026	0.084	0.077	0.0006^*^
Thalamus	*r*=	0.060	0.354	0.413	0.041	0.325	0.283	0.167	0.367	0.417
	*p*=	0.751	0.055	0.023	0.829	0.079	0.130	0.379	0.046	0.022
**CEREBELLAR DIFFUSIVITY**										
Crus I	*r*=	0.235	**0.564**	0.461	0.218	0.370	0.455	0.309	**0.580**	**0.633**
	*p*=	0.212	**0.001**	0.010	0.248	0.044	0.012	0.096	**0.0008**	0.0002^*^
Crus II	*r*=	0.415	**0.583**	0.412	0.375	0.324	**0.556**	0.425	**0.603**	**0.639**
	*p*=	0.023	**0.0007**	0.024	0.041	0.080	**0.0014**	0.019	**0.0004**	0.0001^*^
Anterior vermis	*r*=	0.185	**0.601**	**0.521**	−0.030	0.230	0.408	−0.026	0.484	0.431
	*p*=	0.327	**0.0004**	**0.003**	0.874	0.221	0.025	0.893	0.007	0.017
Posterior vermis	*r*=	0.224	0.278	**0.542**	0.279	−0.028	**0.538**	0.231	0.175	0.444
	*p*=	0.233	0.137	**0.002**	0.135	0.882	**0.0022**	0.219	0.355	0.014
Inferior vermis	*r*=	0.209	0.221	0.016	0.171	0.335	0.040	0.242	0.370	0.258
	*p*=	0.267	0.241	0.934	0.367	0.070	0.833	0.197	0.044	0.169

*Alpha = 0.05 for 17 comparisons requires p ≤ 0.003, noted in bold font*.

* *Apparently graded correlational strength*.

**Figure 3:**
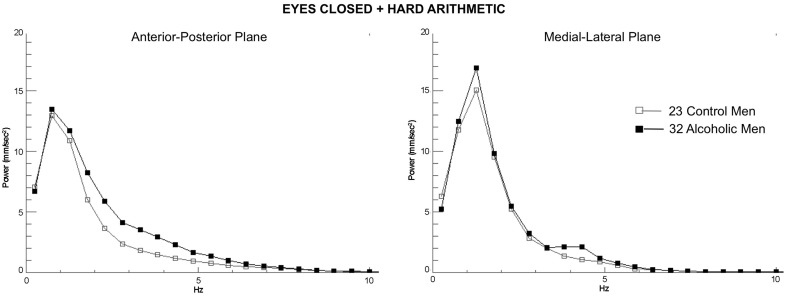
**Group average spectra from the frequency analysis of center-of-pressure sway path velocity (distance/time), presented in ~0.5 Hz frequency bins for the most difficult test condition (eyes closed, hard arithmetic) for the 32 alcoholic men (black lines and filled circles) and 23 control men (gray lines and squares)**. Spectra from the anterior-posterior plane and the medial-lateral plane are plotted separately.

Eight MD measures (internal capsule, splenium, superior cingulum, precentral, middle, and inferior frontal gyri, and Crus I and II) showed a step-wise increase in the magnitude of the correlations with 5–7 Hz tremor velocity (eyes closed) from no task to easy arithmetic to hard arithmetic (Table 2). Exploratory multiple regressions indicated that regional MD measures were consistently better predictors of AP-ML tremor with eyes closed than eyes open, for the 5–7 Hz over lower frequency tremor, and for the harder than easier arithmetic condition. Consequently, to minimize the number of comparisons, our primary DTI-tremor relations focused on path length and tremor measured while engaged in the hard arithmetic task with eyes closed (Figure 3).

#### Sway path length

Neither the control men or women nor the alcoholic women showed significant correlations between sway path length and regional MD. For the alcoholic men, a few modest correlations emerged between regional diffusivity and balance metrics (Table 2), but none was significant after correcting for multiple comparisons (family-wise Bonferroni correction for 17 comparison with a = 0.05 required *p* ≤.003).

#### 2–5/0–2 Hz tremor

**Figure 4:**
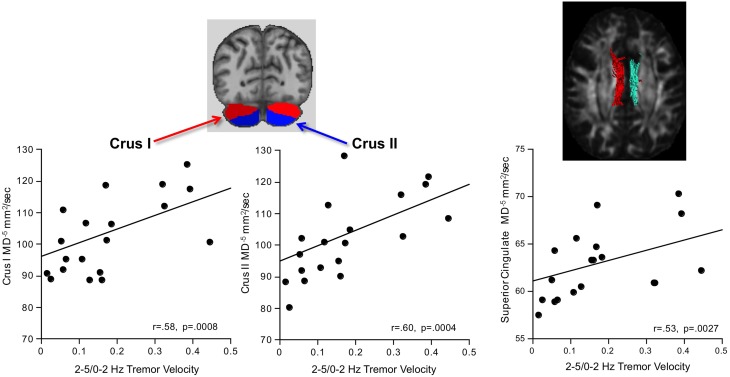
**In alcoholic men, greater 2–5/0–2 Hz tremor velocity correlated with higher MD in the cerebellar Crus I and II**.

Greater AP-ML tremor velocity correlated significantly with higher diffusivity in the superior cingulum, Crus I, and Crus II (Table 2). Multiple regression analysis revealed that MD as a predictor of tremor velocity accounted for 38.9% of the adjusted variance (*p* = 0.0012), where superior cingulate MD made a greater independent contribution to the variance (*p* = 0.08) than MD in either cerebellar region (p > 0.26) (Figure 4). Entering mean 2-point discrimination scores together with superior cingulate MD into a multiple regression accounted for 30.5% of the variance (*p* = 0.0058), with greater unique contribution from MD (*p* = 0.0094) than 2-point discrimination performance (*p* = 0.09).

#### 5-7 Hz tremor

**Figure 5:**
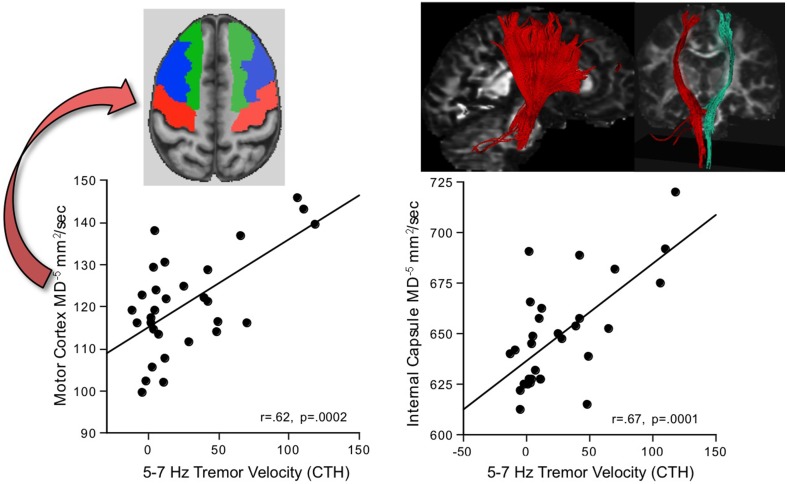
**In alcoholic men, greater 5–7 Hz tremor velocity correlated with higher MD in the motor cortex and internal capsule**.

Greater AP-ML tremor velocity in this frequency band correlated significantly with higher MD in 9 of the 17 regions examined (Table 2): internal capsule, splenium, superior cingulum, four frontal gyri, Crus I, and Crus II. Although entering all 9 MD measures as predictors of tremor accounted for 50.9% (*p* = 0.003) of the variance, a follow-up multiple regression identified major contributions simply from MD in motor cortex (*p* = 0.032) and internal capsule (*p* = 0.0075), together accounting for 49.9% (*p* = 0.0001) of the adjusted variance. Further analysis included 2-point discrimination scores along with motor cortex and internal capsule MD, together accounting for 63.5% (*p* = 0.0001) of the 5–7 Hz tremor variance, and each contributed uniquely to the overall variance (motor cortex MD *p* = 0.0022; internal capsule MD *p* = 0.0934; 2-point discrimination score *p* = 0.0206) (Figure 5).

**Figure 6:**
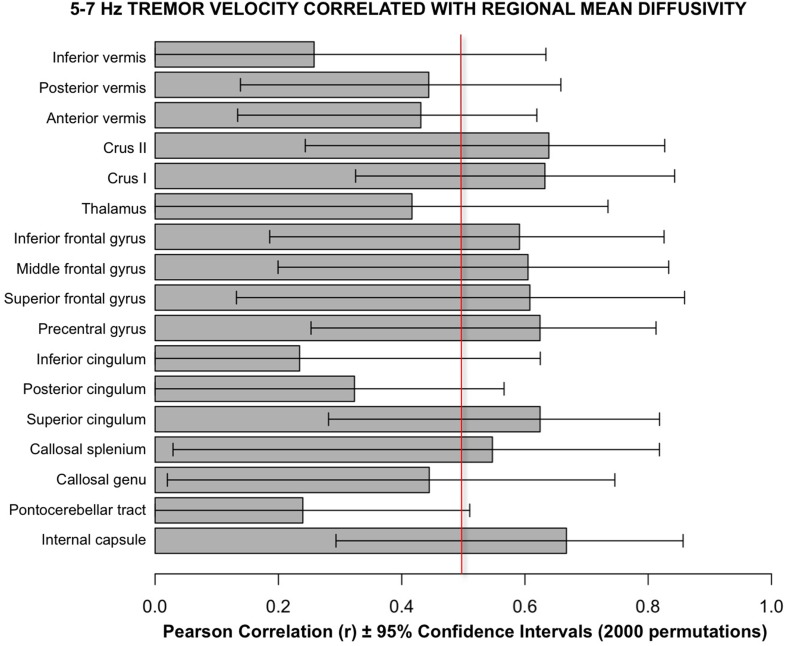
**Bars depict Pearson correlation r for the 5–7 Hz tremor velocity with each of the 17 regional MD measures**. The error bars are the 95% confidence intervals derived from the bootstrapping method using 2000 permutations. The vertical red line marks the approximate level of correlation required to achieve statistical significance after correction for multiple correlations.

Follow-up analyses used a bootstrapping computation conducted in R using 2000 permutations to create confidence intervals for the correlations. The results of bootstrapping supported the Pearson correlations (Figure 6).

## Discussion

Sway path lengths were longer with eyes closed than eyes open and while engaged in the arithmetic, dual task but were not different among the groups. By contrast, truncal tremor, marked by prominences in the 2–5 Hz (normalized to 0–2 Hz) and 5–7 Hz frequency bands, was substantial in the alcoholic men but not in alcoholic women and exacerbated with dual tasking because the fewer the arithmetic errors (indicative of greater attention paid to the arithmetic task), the greater the tremor. With few exceptions, the alcoholics were tested after prolonged sobriety, and no measure of sway or tremor correlated with length of sobriety. Thus, alcoholism-related effects on balance could not be attributed to acute withdrawal. Tremor, greater in the anterior-posterior (AP) than medial-lateral (ML) direction, was related to tissue integrity of an extensive neural system, extending from the cerebellum to frontal cortex and dissociable on the basis of tremor power.

### Relation to tissue integrity of the CNS motor system

The DTI diffusivity metric, MD, a measure of freely diffusing water, assesses brain tissue quality. Abnormally high MD can reflect higher than expected interstitial tissue water or edema, as reported in stroke (Holdsworth et al., 2014), neurodegenerative diseases, and alcoholism (for review Zahr, 2014). Another source of high MD in tissue is partial voluming, that is, inclusion of nontarget signal (typically from sulcal or ventricular cerebrospinal fluid) in the tissue region measured. Although the alcoholics herein did not exhibit abnormally high MD relative to controls, greater tremor in the lower and higher frequency bands did correlate selectively with higher regional MD in alcoholic men. These relations have foundation in lesion studies and disease models, described next.

Monkeys treated with MPTP to model Parkinson's disease exhibited 5–7 Hz arm tremor, which was associated with lesions in the “cerebellar” thalamus and was distinguishable from higher-frequency (>7 Hz) tremor arising from the “pallidal” thalamus (Guehl et al., 2003). To the extent that tremor frequency characterizes compromise of a selective, “tremorgenic” neural system (cf., Brittain and Brown, 2013; Pedrosa et al., 2013) regardless of its peripheral origin, presence of enduring 5–7 Hz postural tremor in long-sober alcoholics may also arise from damage in cerebellar-pontine-thalamic structures (Makris et al., 2008; Sullivan and Pfefferbaum, 2009), frontocerebellar circuitry (Le Berre et al., 2014), or both. Supporting this possibility, greater tremor in the 5–7 Hz frequency band in our alcoholic men correlated selectively with signs of greater tissue compromise in brain regions contributing to motor abilities, including the motor cortex, and corticospinal fiber tracts of the internal capsule, which connect regions enabling sensorimotor integration and information exchange across motor, prefrontal, thalamic, and cerebellar sites. Further, that truncal tremor was greater in the anterior-posterior than medial-lateral axis is similar to symptoms of “frontal lobe ataxia” that can occur with lesions of the cerebellar vermis, thalamus, or frontal cortex (Thompson, 2012).

Differences in tremor characteristics have brain structural underpinnings; indeed, tremors can be classified according to their frequency, amplitude, and anatomical location. For example, Parkinson's Disease is associated with tremor in the 4–6 Hz range, whereas essential tremor typically occurs between 4 and 8 Hz (Anouti and Koller, 1995). Patients with anterior cerebellar lobe lesions show a 3 Hz postural tremor in the AP direction, whereas those with cerebellar hemisphere lesions cannot be separated from controls (Mauritz et al., 1979; Diener et al., 1984), but tremor in the 3–10 Hz range has been associated with cerebellar pathology of various etiologies (Brown et al., 1997).

We used the ratio of 2–5/0–2 Hz to enable comparison of our results in chronic alcoholics, whose cerebellar damage would likely be modest, with results in the patients of the Bahol et al. studies (i.e., Baloh et al., 1994, 1998), who had clinically diagnosed olivopontocerebellar atrophy or isolated cerebellar atrophy and severe gait and balance impairment without visual-vestibular deficits. In the Baloh study, the 2–5/0–2 Hz ratio tremor was observed in patients with cerebellar atrophy but not in those with bilateral vestibular impairment under test conditions of static balance with eyes open (Baloh et al., 1998). The ratio was even greater in the cerebellar group with eyes closed, although 3 of 10 patients in the vestibular group showed such tremor. Alcoholics in our study had no clinical evidence of tremor other than that detected with quantitative measures afforded by force plate analysis; nonetheless, our alcoholic men exhibited enhanced 2–5/0–2 Hz ratio tremor, which was similar to that reported in the severely affected patients with cerebellar lesions reported by Baloh et al.

The constellation of neural sites observed as correlates of alcoholism-related tremor in both power prominences identified herein is consistent with results from the DTI study of Klein et al. (2012), who tracked a network of structures from the ventral thalamic sites that relieved tremor through deep brain stimulation. That network included the precentral gyrus, primary and supplementary motor areas, frontal gyri, anterior cingulum, and the cerebellar hemispheres. These findings also comport with a DTI study of essential tremor that reported abnormally high diffusivity in far-reaching sites, including frontoparietal white matter, anterior internal capsule, thalamus, brain stem, and cerebellar hemispheres and peduncles (Saini et al., 2012).

### Interaction of postural stability with cognitive challenge

Engagement in a cognitively challenging task elicited a subtle abnormality, physiological truncal tremor, while alcoholic men maintained upright posture. Although path length was little affected, tremor was exacerbated while engaged in solving multi-step mental arithmetic. Another study (Pellecchia, 2003) found that engaging in a cognitive task concurrent with quiet standing resulted in increased sway, which was even greater when the concurrent task required information reduction, similar to that required in the difficult arithmetic task used herein. Further, performance by elderly men and women on the Walk While Talking test was poorer with a difficult task (saying every other letter of the alphabet) compared with a simpler task (saying the alphabet) (Verghese et al., 2002). Most relevantly, our results comport with the blood flow study conducted in healthy young adults who exhibited graded frontal brain activation, observed with fNIRS, depending on level of difficulty of the cognitive task engaged in while standing still or walking (Mirelman et al., 2014). Our findings extend the fNIRS study by identifying a physiological outcome in tremor elicited while standing and performing a difficult cognitive task and by identifying extensive brain networks from cerebellum to frontal cortex as underlying the tremor prominences observed with cognitive-motor dual tasking.

An early position held that engagement in higher-order cognition could not intrude on motor performance required for quiet standing, which was considered to be under automatic motor control (for review, Woollacott and Shumway-Cook, 2002). Nonetheless, we found that cognitive tasks can interact with postural stability and that neural sources of these actions and interactions involved both cerebellar and supratentorial brain motor and cognitive systems, including ones that share connections. A functional MRI study revealed a similar network of regions involving the inferior frontal gyrus, middle cerebellar cortex, and thalamus that were invoked while young healthy adults engaged in mental arithmetic (Menon et al., 2000). All nodes of this circuitry have been implicated in motor control [e.g., postural stability (Victor et al., 1989; Diener and Dichgans, 1992; Sullivan et al., 2006), motor preparation (Diener et al., 1989), and articulation (Spencer and Slocomb, 2007; Timmann et al., 2008)] and cognition, including working memory (e.g., Desmond et al., 1997; Schmahmann, 1997; Chanraud et al., 2010), and are commonly disturbed in long-term chronic alcoholism (for reviews, Sullivan and Pfefferbaum, 2005; Oscar-Berman and Marinkovic, 2007). One caveat considers the possible role of articulation in exacerbating tremor. Recognizing the “dose” effect of greater tremor with the more complex, mental arithmetic task, it could be argued that articulation compounded the difficult cognitive challenge and resulted in greater tremor velocity than with the easier cognitive challenge (cf., Dault et al., 2003).

### Sensory status and tremor

Sensory testing was essentially normal in the alcoholic men and women and showed at most a modest relation to tremor. When considered in the context of the DTI brain measures, 2-point discrimination performance did contribute to the overall variance of the 5–7 Hz velocity of truncal tremor, suggesting an interaction of peripheral sensory system condition with a centrally modulated, physiological response of tremor.

## Conclusion

Enhanced physiological resonance (i.e., tremor) in quiet standing is measurable in alcoholic men and endures beyond acute alcohol withdrawal. Elevated tremor, notable in the anterior-posterior axis in two frequency bands, can be detectable during quiet standing even in the absence of excessive sway path length. Excessive 2–5 Hz tremor velocity was related to higher diffusivity in tissue of the cerebellar hemispheres and fibers of the superior cingulate bundle. The 5–7 Hz tremor velocity was correlated with tissue integrity of a network of frontocerebellar sites and internal capsule, a principal connection for these sites. These far-reaching systems associated with enhanced truncal tremor intersect with neural systems underlying cognition, thereby providing avenues for interference observed as exacerbated tremor by engaging in a concurrent, cognitively challenging, task. We speculate that these different tremor prominences identified in chronic alcoholism, where the lower frequency is associated with compromise of brainstem systems and the higher frequency is associated with compromise of brainstem plus supratentorial systems, is consistent with a model of tremor generation based on investigations of Wilson's Disease (Sudmeyer et al., 2006). In that study, tremor in the 4–6 Hz range was associated with oscillatory coherence determined from EEG scalp recordings between cerebellum-thalamus (but not motor cortex), whereas 15–33 Hz tremor was associated with coherence between thalamus and cortical areas, including primary sensory and motor cortices, premotor cortex, and posterior parietal cortex. Taken together, these observations suggest a progression of tremor power generated by (or at least associated with) activity in lower to higher brainstem to cortical circuitry.

Within the context of alcohol dependence, neuroimaging studies report widespread brain tissue volume deficits, but with a predilection for the prefrontal cortex and the cerebellum (e.g., Makris et al., 2008; Le Berre et al., 2014), thalamus (De Bellis et al., 2005; Sullivan and Pfefferbaum, 2009), pons (Pfefferbaum et al., 2002; Sullivan and Pfefferbaum, 2009), and cerebellar peduncles (Chanraud et al., 2007; Mechtcheriakov et al., 2007). These sites have the potential of representing “tremorgenic networks” (Brittain and Brown, 2013; Pedrosa et al., 2013) that when disturbed contribute to postural instability in the recovering alcoholic and potentially put them at heightened risk for falling.

## Author contributions

All authors participated in the analysis and interpretation of the data and the writing of the manuscript. All authors also approved the final version submitted for publication and are responsible for the integrity of the data and analysis herein.

### Conflict of interest statement

The authors declare that the research was conducted in the absence of any commercial or financial relationships that could be construed as a potential conflict of interest.
